# The Effects of Maturation and Dyslexia Risk on Neural Speech‐Sound Encoding and Discrimination at Preschool Stage

**DOI:** 10.1111/ejn.70450

**Published:** 2026-03-08

**Authors:** Sergio Navarrete‐Arroyo, Peixin Nie, Paula Virtala, Teija Kujala

**Affiliations:** ^1^ Cognitive Brain Research Unit, Department of Psychology, Faculty of Medicine University of Helsinki Helsinki Finland; ^2^ Finnish Centre of Excellence in Music, Mind, Body and Brain, Department of Psychology, Faculty of Medicine University of Helsinki Helsinki Finland; ^3^ Finnish Centre of Excellence in Music, Mind, Body and Brain, Faculty of Educational Sciences University of Helsinki Helsinki Finland

**Keywords:** developmental dyslexia, event‐related potentials, maturation, mismatch negativity, neural speech processing, preschool stage

## Abstract

Event‐related potentials (ERPs), and particularly change‐elicited mismatch responses (MMRs), are valuable tools for assessing early speech processing and promising markers of dyslexia risk. Yet, their maturation during the preschool stage remains poorly characterized. We determined the typical elicitation of ERPs and MMRs to speech sounds at preschool age (4–5 years), their maturation from early childhood (28 months) to preschool age, and the impact of dyslexia risk on them. To this end, we recorded obligatory ERPs to a repeating pseudoword and MMRs to five deviances at 4–5 years and compared them with a previously reported follow‐up at 28 months, in subgroups with versus without dyslexia risk in a large sample (*n* ~ 150). In the full sample, including both control and at‐risk children, the 4‐ to 5‐year obligatory ERPs showed a P1–N2 pattern, while the MMRs included a mismatch negativity (MMN) followed by a late discriminative negativity (LDN). From 28 months to 4–5 years, P1 amplitude increased and latency decreased, whereas the N2 amplitude increased. MMN and LDN amplitudes increased and LDN latency decreased with age, whereas a positive MMR reported at 28 months was no longer evident at 4–5 years. Crucially, at‐risk children exhibited reduced MMN amplitudes at 4–5 years across deviants, suggesting deficient speech discrimination. Changes from 28 months to 4–5 years were similar in both subgroups. These findings establish a solid description of typical/atypical neural speech‐sound processing during preschool years, serving as a reference for future studies including interventions or clinical groups.

## Graphical abstract

Event‐related potentials (ERPs) to a repeating pseudoword and mismatch responses (MMRs) to five deviances were recorded at 4–5 years and compared with a previously reported follow‐up at 28 months, in subgroups with versus without familial dyslexia risk (*n* ~ 150). P1–N2 and MMN–LDN components increased in amplitude and decreased in latency with age, whereas the P‐MMR was no longer evident at 4–5 years. At‐risk children showed reduced MMN amplitudes at 4–5 years, suggesting deficient speech discrimination in dyslexia risk.

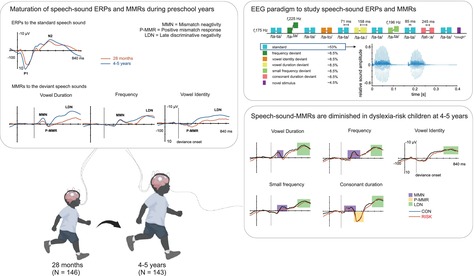

**Abbreviations**
EEG: electroencephalogram
ERP: event‐related potential
ICA: independent component analysis
LDN: late discriminative negativity
MMN: mismatch negativity
MMRs: mismatch responses
P‐MMR: positive mismatch response
ROI: region of interest

## 1 Introduction

Fluent language and literacy are among the most important abilities that contribute to success in school, work life, and interpersonal relationships (Moretti et al. 2013). The early development of efficient auditory and speech processing abilities is essential for this, and difficulties in these domains are known to be associated with later learning disorders (Ozernov‐Palchik and Gaab 2016). Among these, developmental dyslexia (henceforth, dyslexia) is the most prevalent learning disorder. Dyslexia shows moderate heritability (Peterson and Pennington 2015; Kere 2011, 2014) and stems primarily from deficits in native‐language phoneme processing (the phonological deficit theory; Peterson and Pennington 2015; Eden et al. 2016; Vellutino et al. 2004). In addition, it is typically diagnosed during school years, when children exhibit clear difficulties with reading‐skill acquisition (Catts et al. 2016). However, identifying early neural markers of typical/atypical phonological processing could advance our understanding of language development trajectories, as well as the emergence of dyslexia risk before literacy difficulties arise.

Recording auditory event‐related potentials (ERPs) provides a valuable tool for this purpose, as it is noninvasive and does not rely on verbal or motor responses, attention, or motivational factors (Kushnerenko et al. 2013; Hoehl and Wahl 2012; Thierry 2005). Moreover, auditory and speech‐elicited ERPs have been shown to differ, at the group level, between individuals with or without dyslexia (Kujala and Näätänen 2001; Ozernov‐Palchik and Gaab 2016), providing valuable insights into the neural processes underlying reading difficulties and their development. Repetitive auditory stimuli elicit the so‐called obligatory ERPs, while deviations from this pattern generate the mismatch negativity (MMN), indicative of sound discrimination (Csibra et al. 2008; Näätänen et al. 2019). In infants and young children, however, change‐detection responses often differ from the adult‐like MMN and are typically referred to as mismatch responses (MMRs). Unlike the MMN, MMRs may be either negative or positive going in polarity, which is generally attributed to developmental differences in the underlying neural generators and cortical maturation (Csibra et al. 2008; Kushnerenko et al. 2002; Virtala et al. 2022). While ERPs and their atypicalities in dyslexia have been studied in early infancy (Volkmer and Schulte‐Körne 2018) and later childhood (Stefanics et al. 2011; Lachmann et al. 2005), studies focusing on the preschool stage—the period between infancy and the start of primary school (approximately 2–6 years)—remain limited. This study, encompassed in the longitudinal DyslexiaBaby study, addresses this gap by examining the elicitation of obligatory ERPs and MMRs to speech stimuli at 4–5 years, as well as the influence of familial risk for dyslexia on these responses. Additionally, by utilizing previously reported data from the same sample at 28 months of age (Virtala et al. 2022), the current study gives an opportunity to inspect the maturation of both the obligatory ERPs and MMRs from 28 months to 4–5 years, as well as the effects of dyslexia risk on this maturation.

### 1.1 Auditory ERPs and Their Development During the Preschool Stage

In infants and young children, the obligatory ERP complex typically consists of a broad positivity (P1) followed by a negativity (N2; Ponton et al. 2002; Shafer et al. 2015; Choudhury and Benasich 2011; Kushnerenko et al. 2002). These responses show rapid maturation during infancy, with increasing amplitudes and decreasing latencies, as demonstrated in several longitudinal studies (Shafer et al. 2015; Choudhury and Benasich 2011; Kushnerenko et al. 2002), including recent findings from the DyslexiaBaby project (Navarrete‐Arroyo et al. 2025, using speech sounds). This trajectory extends beyond infancy: P1 and N2 reach their maximum amplitudes in early childhood (6–8 years of age) and then diminish from 9–10 years onwards, with the N2 becoming almost absent in adults (Shafer et al. 2015; Ponton et al. 2002; Sussman et al. 2008).

The developmental trajectory of the MMR includes emerging components and changes in response polarities during the first months and years of life. Infant studies report a prevalent positivity (positive mismatch response [P‐MMR]) that is preceded by an emerging negative response (MMN) and followed by another late negativity (late discriminative negativity [LDN]; Kushnerenko et al. 2002; Čeponienė et al. 2000; Fellman et al. 2004). The P‐MMR is most prominent at birth but decreases in amplitude after 6 months, while both the MMN and LDN grow in amplitude with age (Kushnerenko et al. 2002; Čeponienė et al. 2000; Fellman et al. 2004; Virtala et al. 2022). Notably, this developmental pattern was replicated within the DyslexiaBaby project (Virtala et al. 2022), which tracked the elicitation and maturation of speech‐elicited MMRs from birth to 28 months in a large sample of children. Around 6 years of age, there is a shift toward more mature profiles, characterized by decreasing P‐MMR and increasing MMN amplitudes in response to both speech (Yu et al. 2019; Lee et al. 2012; Linnavalli et al. 2018) and nonspeech sounds (Choudhury and Benasich 2011; Morr et al. 2002).

Despite the considerable number of children's ERP studies available, most have focused on infancy or school age, leaving the elicitation and maturation of these responses during the preschool years relatively unclear (Themas et al. 2023). While age ranges for these developmental phases vary across studies, we here define infancy as approximately 0–2 years, preschool age as approximately 2–6 years, and school age as approximately 6–12 years. Yet the preschool years are a critical developmental stage, as phonological processing abilities during this period are believed to shape later language and literacy outcomes (Scarborough 1990; Torppa et al. 2010). The authors are aware of three cross‐sectional (Shafer et al. 2010; Maurer et al. 2009; Linnavalli et al. 2018) and two longitudinal (Shafer et al. 2015; Yu et al. 2019) studies investigating the obligatory ERPs at the preschool stage. The results show that P1 and N2 remain the most prominent obligatory responses to speech sounds in preschoolers. Findings on MMRs are more variable, with cross‐sectional studies reporting a P‐MMR followed by a MMN (Shafer et al. 2010), others only a P‐MMR (Liu et al. 2014), only an MMN (Lovio et al. 2009), and still others observing an additional LDN (Maurer et al. 2009; Linnavalli et al. 2018). These inconsistencies may result from methodological variability, such as differences in stimulus type and presentation rate. They may also reflect limited statistical power due to small sample sizes (only one study with over 50 participants), coupled with the often‐suboptimal quality of child EEG data. Moreover, those two studies which longitudinally tracked the maturation of ERPs to speech sounds from infancy through the preschool stage were also constrained by small sample sizes (Shafer et al. 2015; Yu et al. 2019, 19–39 participants). Taken together, there is a need for longitudinal studies employing age‐appropriate paradigms and large samples to clearly characterize the elicitation and maturation of auditory ERPs in the preschool stage. Such studies are crucial for establishing a solid foundation that can inform future research on the effects of interventions and auditory processing disorders in early childhood.

### 1.2 ERPs and Deficient Auditory Processing Abilities in Dyslexia

Atypical auditory and speech processing in individuals at familial risk for dyslexia is reflected in altered ERP responses. The most consistent findings concern auditory discrimination, with both positive and negative MMRs often being reduced or absent in infants and young children at risk for the disorder (for a review, see Volkmer and Schulte‐Körne 2018). Furthermore, atypical hemispheric lateralization of MMRs in individuals at risk for dyslexia has been reported in response to both speech and nonspeech sounds. For example, some studies have found group differences only in the left hemisphere (Benasich et al. 2006; Choudhury and Benasich 2011; Leppänen et al. 2002), whereas others have reported right‐lateralized MMRs in the risk group (van Leeuwen et al. 2006; Kuuluvainen, Alku, et al. 2016). Such findings are in line with the suggested more prominent role of the left than the right hemisphere in speech processing (Hickok and Poeppel 2007), as well as with structural neural deficits in the left temporal areas of individuals with dyslexia (Kujala et al. 2021; Sihvonen et al. 2021). Findings on obligatory ERPs are more variable, with few studies reporting reduced and/or atypically lateralized P1/N2 to speech sounds in infants (van Herten et al. 2008; Molfese 2000) and children (Kuuluvainen, Leminen, and Kujala 2016) at risk for dyslexia.

Previous investigations within the DyslexiaBaby cohort examined dyslexia‐risk effects on speech processing between birth and 28 months. At birth, 6 months, and 28 months, infants at risk for dyslexia showed enlarged P‐MMRs to salient vowel duration changes but reduced P‐MMRs to subtler vowel identity changes in pseudoword streams (Virtala et al. 2022). These results align with earlier findings of atypical speech‐sound discrimination in dyslexia risk and suggest that less salient deviants may still be processed immaturely at 28 months (Cheng et al. 2013, 2015). Thus, in at‐risk infants, a small P‐MMR to vowel identity changes may reflect inaccurate discrimination of subtle contrasts, while a large P‐MMR to vowel duration changes could indicate immature processing of salient contrasts. For the obligatory ERPs to speech sounds, no major effects of dyslexia risk were observed at the same three age points, the only observation being an enlarged N2 in the risk group at 6 months of age (Navarrete‐Arroyo et al. 2025). Furthermore, no significant hemispheric differences in ERPs or MMRs to speech stimuli were observed between groups in any of the DyslexiaBaby studies. This provides limited support for previous reports of atypical hemispheric lateralization of neural speech processing in dyslexia risk.

Based on the reviewed literature, two issues are important when assessing auditory deficits in dyslexic children. First, auditory deficits associated with dyslexia appear to be the most evident at birth and then attenuate or become compensated during early development (Galaburda et al. 2006; Hämäläinen et al. 2013). Consistent with this, the DyslexiaBaby study found that familial dyslexia risk affected MMRs in infants (Virtala et al. 2022), while no such deficits were observed in dyslexic adults studied with the same paradigm (Thiede et al. 2020). However, the deficits were still visible at 28 months of age. Whether they persist throughout the preschool years or attenuate with age will be determined in the present study. Second, the choice of the auditory stimuli and paradigm is crucial, as the acoustic features must be developmentally appropriate to reveal potential deficits. In order to pinpoint auditory deficits in dyslexia, the acoustic difference between the standard and deviants should be sufficiently small (Baldeweg et al. 1999), and the stimulus sequences should contain variation (e.g., Kujala et al. 2006). Therefore, small adjustments were made to the DyslexiaBaby stimulus paradigm to ensure its validity for investigating auditory deficits in dyslexia risk in the current study (Kujala and Näätänen 2010; Bishop and McArthur 2005; Kujala et al. 2006).

### 1.3 Research Questions and Hypotheses

The main aim of this study was to determine the typical morphology of obligatory ERPs and MMRs to speech sounds at the age of 4–5 years in the large longitudinal DyslexiaBaby sample. To do that, we examined obligatory ERPs elicited by a pseudoword and MMRs elicited by five deviants (vowel duration, frequency, vowel identity, small frequency, and consonant duration). Based on the above‐reviewed literature, we expected the obligatory ERP waveform to be characterized by a prominent P1–N2 complex and a discrimination response pattern dominated by MMN and LDN components. While a P‐MMR is expected to be greatly attenuated by this age, it may be observed particularly in response to the acoustically least salient deviants (i.e., the consonant duration, small frequency, and vowel identity deviants). Additionally, the present study aimed to longitudinally examine the developmental trajectory of the speech‐elicited obligatory ERPs and MMRs from 28 months to 4–5 years. As these responses and the impact of dyslexia on them at 28 months have been reported in our previous publications (Virtala et al. 2022; Navarrete‐Arroyo et al. 2025), the sole focus will be on investigating the maturational changes from 28 months to 4–5 years. According to previous reports and earlier trends observed within the DyslexiaBaby study, we hypothesized that the P1 and the N2 increase in amplitude and decrease in latency with increasing age. The MMRs, in turn, were expected to mature as follows: The P‐MMR was predicted to become smaller or absent with increasing age, while the MMN and LDN were predicted to increase in amplitude and possibly decrease in latency.

We also aimed to examine differences between children with or without a familial risk for dyslexia in the elicitation and properties of obligatory ERPs and MMRs at 4–5 years of age, as well as in their maturation from 28 months to 4–5 years. Based on mixed findings in previous studies on the present dataset and other studies, it is unclear whether the obligatory ERPs will demonstrate effects of dyslexia risk (Navarrete‐Arroyo et al. 2025). However, based on previous findings, we hypothesized that the MMNs and/or LDNs would be reduced by dyslexia risk. If P‐MMRs are elicited, we expect them to be larger in the at‐risk group as a sign of immature neural discrimination of the speech stimuli. Furthermore, the five different deviant types might overall elicit smaller MMNs and/or LDNs in dyslexia‐risk than no‐risk children. However, there might also be group differences distinctly for some deviant types. For instance, the small frequency deviant incorporated into the paradigm used in the current study might elicit more prominent MMR differences between children at risk and controls. As for hemispheric lateralization, while previous studies might lead to the expectation of atypical patterns in the at‐risk group, the largely null results reported at earlier time points temper such expectations for this sample (Virtala et al. 2022).

## 2 Methods

### 2.1 Participants

This study includes children from the longitudinal DyslexiaBaby project (2014–present), assessed at two developmental time points: 28 months and 4–5 years (Table 1). The original cohort comprised 210 infants recruited prenatally or neonatally through multiple channels: media outreach, social media campaigns and partnerships with maternity clinics. To be enrolled in the study, children had to be born healthy (5‐min Apgar score of 7–10) and full‐term (at least 37 weeks and birth weight of at least 2500 g). In addition, they had no reported hearing problems (tested through evoked otoacoustic emissions) and had Finnish as (one of) their native language(s). A detailed description of the testing protocol, as well as of the inclusion criteria, can be found in Virtala et al. (2022).

**TABLE 1:** Background information and sample sizes in the whole sample (in bold) and in the risk (RISK) and control (CON) subgroups at 28 months (28mo) and 4–5 years (4–5yo): amounts of the DyslexiaBaby dyslexia‐risk and con(trol) participants; amounts of the DyslexiaBaby intervention (int1, int2), no‐int(ervention), and con(trol) group participants; gender distributions; socio‐economic status as indicated by amounts of high and low edu(cation); and electroencephalogram (EEG) recording age (in days or months). Participants in the “low edu” group had no parents with higher education (tertiary education leading to an academic degree).

	28mo			4–5yo		
	Whole sample (*N* = 146)	CON (*N* = 32)	RISK (*N* = 28)	Whole sample (*N* = 143)	CON (*N* = 32)	RISK (*N* = 30)
Dyslexia risk/con	**114/32**			**111/32**		
int1/int2/no‐int/con	**37/37/40/32**			**35/37/39/32**		
High/low edu	**125/21**	29/3	26/2	**118/25**	28/4	27/3
Female/male	**67/79**	14/18	11/17	**70/73**	15/17	12/18
Age, months (SD)	**28.1 (0.4)**	28.3 (0.4)	28.0 (0.4)	58.16 (2.31)	57.78 (2.82)	58.08 (2.41)

*Note:* Due to the COVID‐19 pandemic, there were delays in the data collection at 4–5 years. As a result, there was greater variability in the ages of participants at the time of measurement compared with earlier assessments of the DyslexiaBaby study.

Within the sample, 3/4 of the children had an elevated risk of dyslexia (risk group, 114 of 146 at 28 months, 111 of 146 at 4–5 years). In these children, at least one of the biological parents had a confirmed diagnosis of dyslexia or was deemed a compensated dyslexic. For a confirmed diagnosis of dyslexia, the parent had to report clear reading difficulties that started in childhood and either have a recent (< 5 years) diagnostic statement from a health care professional or, at study enrolment, demonstrate below‐norm performance of at least two standard deviations in reading or writing speed or accuracy in at least two out of four subtests of a dyslexia test battery (Nevala et al. 2006). In some cases, parents reported significant reading or writing difficulties during childhood, along with a family history of dyslexia, but did not fully meet the diagnostic criteria. These parents were classified as compensated dyslexics. Additionally, 2/3 (74 of 114 at 28 months, 72 of 111 at 4–5 years) of the risk group children had participated in one of two music listening interventions during the first 6 months of life (for a description of the intervention, see Virtala and Partanen 2018). While the intervention showed positive effects on speech‐sound discrimination (enhanced MMRs) at 6 months, directly after the intervention, the effects were no longer seen at 28 months (Virtala et al. 2023).

To study possible effects of dyslexia risk on the obligatory ERP and MMR elicitation and maturation in more detail, an additional high‐risk subsample was taken from the risk group (Table 1). The high‐risk group consisted of those children of the risk group who did not participate in the music intervention (no‐int group in Table 1) as that may affect their ERPs, excluding those with parents classified as compensated dyslexics. A similar protocol has been adopted in previous studies from the DyslexiaBaby project (Virtala et al. 2022; Navarrete‐Arroyo et al. 2025; Thiede et al. 2019). The control children (con in Table 1) were all included in the control group of the present study.

Obligatory ERP and MMR maturation was investigated in the whole sample, including all children with usable electroencephalographic data (without excessive artifacts, no more than five bad channels based on visual inspection, and enough trials; see Section 2.4) from at least one measurement point. Children were excluded from the analysis due to the following reasons: failure to meet inclusion criteria (18 children), families withdrawing from the study (4), contact lost (1), children with no EEG data at 28 month (27), EEG‐data‐quality issues at 28 months (14), children with no EEG data at 4–5 years (37) and EEG‐data‐quality issues at 4–5 years (7). Children with poor EEG‐data quality at one time point were excluded from the analysis for that specific time point, but remained included at the other time point if their data met quality standards. The final samples consisted of 146 children with usable EEG data at 28 months and 143 children with complete data at 4–5 years (Table 1). In total, 119 children had complete data at both time points.

One or both parents of the participants included in the study gave written informed consent to participate. The DyslexiaBaby study was approved by The Ethics Committee for Gynecology and Obstetrics, Pediatrics and Psychiatry of the Hospital District of Helsinki and Uusimaa. In addition, it was performed in compliance with the Declaration of Helsinki.

### 2.2 Experimental Stimuli and Paradigm

#### 2.2.1 Stimuli

The experimental stimuli (first used Pakarinen et al. 2014) consisted of a bisyllabic pseudoword /tata/ (original stimulus) and its variants, all uttered by a female native Finnish speaker. The original stimulus was naturally spoken with stress on the first syllable and had a total duration of 300 ms, of which ~250 ms were audible. The second syllable began at ~168 ms, and the second /a/ started at ~181 ms. There was a pause of approximately 85 ms between syllables. The variants were created by modifying the second syllable of the original stimulus /tata/ using Adobe Audition CS6 (Version 5.0; Adobe Systems Inc.) and Praat (Version 5.4.01; Boersma and Weenik 2013). The following five variants (deviants) were created.

In the vowel duration deviant, the total length of the second syllable was extended from 71 to 158 ms. In the frequency deviant, the fundamental frequency of the second syllable was lifted from 175 to 225 Hz. The vowel identity deviant was created by replacing the second /a/ with a naturally uttered /o/ with the same start, fundamental frequency, and duration as the original stimulus.

In the small frequency deviant, the fundamental frequency of the second syllable was lifted from 175 to 196 Hz. Finally, the consonant duration deviant was created by extending the pause between syllables until approximately 245 ms, 160 ms longer than in the original /tata/ stimulus.

#### 2.2.2 Paradigms

At both measurement points, the original /tata/ stimulus and its variants were presented in a multifeature oddball paradigm (Näätänen et al. 2004), where the original /tata/ stimulus served as the repeating standard and its variants served as occasional deviants as described below. Both the 28‐month and 4‐ to 5‐year paradigms also included rare novel human (e.g., sigh, cry, and laugh) and nonhuman (e.g., telephone ring and electric drill) sounds; these stimuli were excluded from the analyses reported here (partially reported in Kujala et al. 2024).

At 28 months, the paradigm included three deviant types (vowel duration, frequency, and vowel identity), implemented in previous measurement points of the DyslexiaBaby study (e.g., Thiede et al. 2019; Kailaheimo‐Lönnqvist et al. 2020; Virtala et al. 2022). The stimuli were presented in four blocks of 472 stimuli (altogether 1325 standards [70.1%], 160 repetitions per each deviant [8.5%], and 85 novel sounds [4.5%]), where the stimuli were presented otherwise in a random order, but so that four standards started each block, and a standard always followed a deviant or novel stimulus.

**FIGURE 1:**
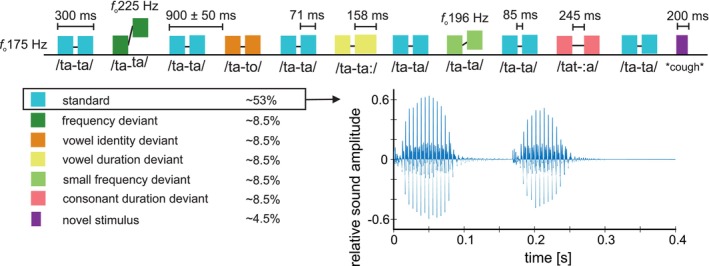
Experimental paradigm and stimuli at 4‐ to 5‐year EEG measurement. Top: Illustration of the experimental paradigm with the stimulus types separated by colors as described in the left bottom corner, with (1) fundamental frequencies (fo, in Hz) separately for the frequency deviants and for the other stimulus types; (2) duration (in ms) separately for the vowel duration deviant, novel stimuli, and the other stimulus types; and (3) intersyllabic gap separately for the consonant duration and for the other stimulus types. Bottom: In the left, stimulus types, their color labels, and their probabilities (in %). In the right, illustration of the sound waveform of the standard /tata/ stimulus, with time (in seconds, s) in the *x* axis and relative sound amplitude in the *y* axis. Illustration for the EEG paradigm at 28 months can be found in Virtala et al. (2022).

At 4–5 years, the set of deviant stimuli closely resembled that used at 28 months but was expanded to increase sensitivity to potential group differences. Two additional deviant types were introduced: small frequency and consonant duration deviants (Figure 1). The same paradigm structure was retained but with a modified stimulus probability. Four blocks of 435–457 stimuli were presented, maintaining an 8.5% probability per deviant and a 4.5% probability for novel sounds but reducing the standard probability to 53%. Each block started with four standards, but after that stimuli were implemented in an “Optimum‐1” sequence, in which deviant/novel sounds were inserted among the standard sounds in a pseudorandom sequence (Näätänen et al. 2004). To reduce the expectancy effects related to the predictability of the stimulus onset, both paradigms used a stimulus‐onset asynchrony of 900 ± 50 ms, alternating randomly in 10‐ms steps.

### 2.3 EEG Recordings

EEG recordings were carried out by a trained research assistant and took approximately 1–2 h, including preparations. They were performed using a BrainProducts Quick‐Amp amplifier (v. 10.08.14; software: BrainVision Recorder 1.20.0801, Brain Products GmbH, Gilching, Germany), with an electrode cap (ActiCap, Brain Products GmbH, Gilching, Germany) with 32 electrodes placed according to the extended international 10/20 system. Stimuli were presented using Presentation 17.2 software (Neurobehavioral Systems Ltd., USA) through two loudspeakers (Genelec speaker) with an intensity of ≈65‐dB SPL. The data were acquired with a 500‐Hz sampling rate and hardware filters at DC–100 Hz. The recordings were conducted after a 2‐h neuropsychological evaluation, on a different day or, rarely, on the same day after a long break, in a soundproof, electrically shielded laboratory at the University of Helsinki (28 months *N* = 131/146, 4–5 years *N* = 128/143) or a similar laboratory at the University of Jyväskylä (28 months *N* = 15/146, 4–5 years *N* = 15/143). During the recordings, the child was instructed not to talk or move. At 28 months, children were sitting on parents' lap placed 160 cm from the speakers, and their attention was directed away from the stimuli by displaying muted cartoons. At the age of 4–5 years, children were sitting on a chair and playing with a tablet computer or watching muted cartoons.

### 2.4 EEG Preprocessing

Before preprocessing, EEG data from those stimulus blocks during which the child fell asleep were excluded from the data. Preprocessing was conducted using Matlab 2017a–2020a (The MathWorks Inc., USA) with Toolboxes EEGLAB 14.0.0b and 2019_0 (Delorme and Makeig 2004) and ERPLAB 7.0.0 (Lopez‐Calderon and Luck 2014).

#### 2.4.1 Preprocessing of EEG Data Recorded at 28 Months

The 28‐month EEG data were initially filtered (0.025–40 Hz) in order to get rid of low‐ and high‐frequency artifacts, thus allowing for visual inspection. During visual inspection, electrodes with flat or continuously noisy signal were marked as “bad.” Peripheral electrodes (T7, T8, Po9, Po10, O1, and O2) with bad data were excluded from the analysis, whereas “bad” central electrodes (F3, Fz, F4, C3, Cz, C4, P3, Pz, P4, FC5, FC1, FC2, FC6, CP5, CP2, CP3, and CP6) were interpolated at a later stage. Additionally, data segments with muscle‐related artifacts were manually excluded from the data. Eye‐movement and heart‐beat artifacts were marked in the data for a later removal stage.

Following visual inspection, EEG data were filtered with a 0.5‐ to 25‐Hz band pass and re‐referenced to the average of two mastoid (LM and RM) and two posterior (P7 and P8) electrodes. After that, spherical interpolation of central bad channels was conducted. Such interpolation process was only performed when absolutely necessary, and strict instructions and limits (a maximum of three electrodes per subject; channels adjacent to each other were not interpolated) were followed to ensure the enhancement of data quality. Following interpolation, eye‐movement and heart‐beat artifacts were corrected using independent component analysis (ICA) using fastica (Hyvarinen 1999) or runica algorithms in EEGLAB. The found independent components were compared with artifacts in the raw data to decide whether they should be omitted from the data. If based on visual inspection the removal of the component was unsatisfactory (e.g., artifact was not diminished or algorithm changed other parts of the data), the component was not removed.

After this, continuous EEG data were segmented into epochs starting at −100 ms and ending 840 ms after stimulus onset, with baseline correction according to the average voltage in the −100‐ to 0‐ms prestimulus interval. Moreover, to minimize artifacts related to eye movements and slow signal drifts, epochs were excluded if they met any of the following criteria: (1) amplitude exceeding ±120 μV at the Fp1 or Fp2 electrodes, (2) signal drift greater than 100 μV, or (3) data points deviating more than ±3 standard deviations from the mean amplitude across all epochs. The remaining epochs were separated by stimulus type for each stimulus block and electrode. Notably, epochs for standard stimuli immediately following a deviant were not included in the standard epochs. After segmentation, epochs of all stimulus blocks of the same stimulus type, separate for each participant, were combined, resulting in one dataset per participant and stimulus type. Finally, data of children with less than 30 accepted epochs for more than one deviant were rejected (28 months *n* = 7). The mean number of accepted trials for each deviant was 78 at 28 months. Subtraction waveforms were calculated separately for each deviant by subtracting the standard response from the deviant one. The baseline correction was shifted to −100 to 0 ms from deviation onset, resulting in a baseline correction window of 125–225 ms for the duration deviant and 80–180 ms for the frequency and vowel identity deviants. Deviant onset was defined as the earliest point at which the deviant stimulus audibly diverged from the standard, rather than the physical onset of the altered segment. Accordingly, for the vowel duration deviant, onset was set at 225 ms—slightly preceding the physical onset of the second /a/ (239 ms)—to reflect the expected timing of duration change detection.

#### 2.4.2 Preprocessing of EEG Data Recorded at 4–5 Years

The preprocessing pipeline for EEG data from 4‐ to 5‐year‐old children followed a similar structure to the 28‐month protocol while incorporating modifications to account for developmental differences in neural activity and artifact profiles. Continuous EEG data were initially bandpass filtered between 0.5 and 40 Hz, with a stricter high‐pass cutoff implemented to better address the increased slow‐wave drift characteristic of older children. Following filtering, electrodes with flat or continuously noisy signal were marked as “bad” for later preprocessing step via visual inspection. Peripheral electrodes (F7, F8, T7, T8, O1, Oz, O2, PO9, and PO10) with bad data were excluded from the analysis, whereas “bad” central electrodes (F3, Fz, F4, FC5, FC1, FC2, FC6, C3, Cz, C4, CP5, CP2, CP3, CP6, P3, Pz, and P4) were interpolated at a later stage.

Following visual rejection and artifact marking, data were segmented into epochs (−100 to 840 ms relative to stimulus onset) and baseline‐corrected using the prestimulus interval (−100 to 0 ms). Epochs with extreme amplitude fluctuations (exceeding ±500 μV) were automatically discarded at this point to remove movement‐related transients. Following this, ICA was conducted using the runica algorithm in EEGLAB. Eye‐related independent components are identified with the IClable plugin (Pion‐Tonachini et al. 2019) in EEGlab using a machine learning algorithm. The independent components labeled with at least 80% probabilities of “Eye movements” component but less than 5% probabilities of “Brain activities” component were removed from the data. Following ICA, a 25‐Hz low‐pass filter was applied to attenuate residual high‐frequency noise, including muscle activity and environmental artifacts. Subsequent processing steps replicated the 28‐month protocol: spherical interpolation of bad channels using identical parameters was conducted, followed by re‐referencing to the average of mastoid and posterior electrodes, and application of a second baseline correction (−100 to 0 ms) to ensure signal stability after re‐referencing.

Epoch rejection applied the same criteria as in the 28‐month analysis, except that standard trials following deviants were retained. This was because in the 4‐ to 5‐year paradigm, every other stimulus was a standard. After epoch rejection, all valid epochs of the same stimulus type were merged for each participant. Participants with fewer than 30 accepted epochs for more than one deviant were excluded from further analysis (*n* = 1). Subtraction waveforms were calculated separately for each deviant by subtracting the standard response from the deviant one. The baseline correction was shifted to −100 to 0 ms from deviation onset, resulting in a baseline correction window of 125–225 ms for the duration deviant and 80–180 ms for the other four deviants. To maintain consistency across conditions, the deviation onset was defined uniformly for all deviants, starting after the first syllable (approximately at the time of an audible deviation from the standard), even though a slightly earlier onset could have been chosen for the consonant duration deviant.

### 2.5 ERP Quantification

ERP peak latencies and mean amplitudes were quantified by searching individual positive or negative polarity peaks from broad time windows and measuring the mean amplitude from narrow time windows centered at these peaks, respectively. For obligatory ERPs, the broad time windows were chosen based on visual inspection of the average waveforms across the whole sample. At both measurement points, the selected time windows, reported in milliseconds after stimulus onset, were 50–250 ms for P1 and 300–500 ms for N2.

For the MMRs, the broad time windows for peak latency and mean amplitude quantification were chosen as follows. Spatiotemporal windows showing significant deviance‐related differences from deviance onset to the end of the epoch (840 ms) were identified using cluster‐based mass permutation tests implemented in the FieldTrip toolbox (Maris and Oostenveld 2007; Oostenveld et al. 2011; Table 2). Such tests were implemented separately for each deviant type and measurement time point. First, time ranges with significant deviant–standard differences (*p* < 0.05) with the same polarity in adjacent time points at two or more neighboring channels were determined. Then, the sum of *t* values for each cluster was computed. The test statistic was defined as the maximum of these sum *t* values. To establish the null distribution for this statistic, 5000 permutations were performed by randomly reassigning condition labels (deviant vs. standard) while maintaining the original trial structure. For each permutation, the test statistic was recalculated. Clusters observed in the actual data were considered statistically significant if their summed *t* values fell above the 97.5th percentile or below the 2.5th percentile of the permutation‐derived null distribution. This analysis included all electrodes except those serving as peripheral channels or reference sites.

**TABLE 2:** Search windows used for mean amplitude calculation of the mismatch negativity (MMN), positive mismatch response (P‐MMR), and late discriminative negativity (LDN) responses at 28 months (28mo) and 4–5 years (4–5yo). Search windows describe the latency windows (in ms from deviance onset) for searching the individual response peaks. Width (in ms, in brackets) indicates the width of the latency window that was centered at the individual response peaks in order to calculate the individual mean amplitudes. The epoch ends at 615 ms from deviance onset for the vowel duration deviant and at 660 ms from deviance onset for the rest of deviants.

Age	MMR	Search windows (width), ms				
		Vowel duration	Frequency	Vowel identity	Small frequency	Consonant duration
28mo	MMN	100–200 (25)	100–300 (45)			
	P‐MMR	115–265 (30)		100–250 (45)		
	LDN	415–615 (45)	460–660 (45)	460–660 (45)		
4–5yo	MMN	65–155 (25)	160–290 (25)		140–290 (30)	100–200 (25)
	P‐MMR					220–320 (25)
	LDN	425–615 (40)	460–700 (45)	380–700 (60)	460–700 (45)	470–700 (40)

Thereafter, individual mean obligatory ERP and MMR amplitudes were extracted separately for each stimulus type and measurement point using the toolboxes EEGlab (Version 14.0.0; Delorme and Makeig 2004) and CBRUPlugin (Version 2.0b) in MATLAB (Release 2018b; The MathWorks Inc., Natick, Massachusetts, USA). In case a peak was not found from the broad time window for some participant, the peak latency for that participant was treated as a missing value, and their mean amplitude was calculated by centering a narrow window at the group‐average peak latency instead.

All obligatory ERP and MMR amplitudes were quantified in a large region of interest (ROI) of 10 electrodes (F3, Fz, F4, C3, Cz, C4, FC1, FC2, FC5, and FC6) chosen in accordance with the fronto‐central predominant scalp distribution of the ERPs (Näätänen et al. 2007; Liégeois‐Chauvel et al. 1994). Furthermore, additional left and right ROI mean amplitudes were quantified from the left‐ and right‐hemisphere electrodes (all large ROI electrodes except for midline Fz and Cz) to study the hemispheric distribution of the responses.

### 2.6 Statistical Analysis

Maturational changes in ERP peak latencies and mean amplitudes were analyzed using linear mixed models (LMMs) in R (Version 4.0.4; RStudio) with the lme4 package (Bates et al. 2015). Measurement age was included as a fixed factor, and subject was modeled as a random factor. The maturation of the obligatory ERPs elicited by the standard stimulus was analyzed separately for P1 and N2. Similarly, separate analyses were conducted for MMN, P‐MMR, and LDN, but when the same response was elicited by several deviants in more than one measurement point, deviants were analyzed together, and deviant was included as a fixed factor in the model (main effect of stimulus not reported). Consequently, the maturation of the small frequency and consonant duration deviants, incorporated only at 4–5 years, was not analyzed. To investigate hemispheric differences in mean amplitudes, the LMMs were repeated with the left and right ROIs, with hemisphere included as a fixed factor in the model. Because hemisphere was not significant in any of the LMMs, it was excluded from the final models.

Statistical significance of the MMRs within the high‐risk and control subgroups at 4–5 years was analyzed with One‐sample *t* tests (Bonferroni corrected for multiple comparisons within age group). Group differences were analyzed for all responses that were statistically significantly elicited by at least one deviant and at least one of the two groups based on the one‐sample *t* tests. Such procedure was not needed for the obligatory ERPs, as they had a higher number of trials, which resulted in more robust responses. In the case of obligatory ERPs, group differences were analyzed with a one‐way ANOVA. In the case of MMRs, when an MMR was significantly elicited in at least one of the two groups, group differences (high risk vs. control) in peak latencies and mean amplitudes were investigated with a repeated‐measures analysis of variance (RM‐ANOVA), with stimulus type as within‐subject factor (if several deviants elicited the same response), or a One‐way ANOVA (if only one deviant elicited the response). To assess differences in the maturation of obligatory ERPs/MMRs, the effect of group was added in the LMMs described above. To investigate hemispheric differences in mean amplitudes between groups, the LMMs and ANOVAs were repeated with the left and right ROIs, with hemisphere included as a fixed/within‐subject's factor in the model.

## 3 Results

### 3.1 Obligatory ERPs in the Whole Sample at the Age of 4–5 Years

**FIGURE 2:**
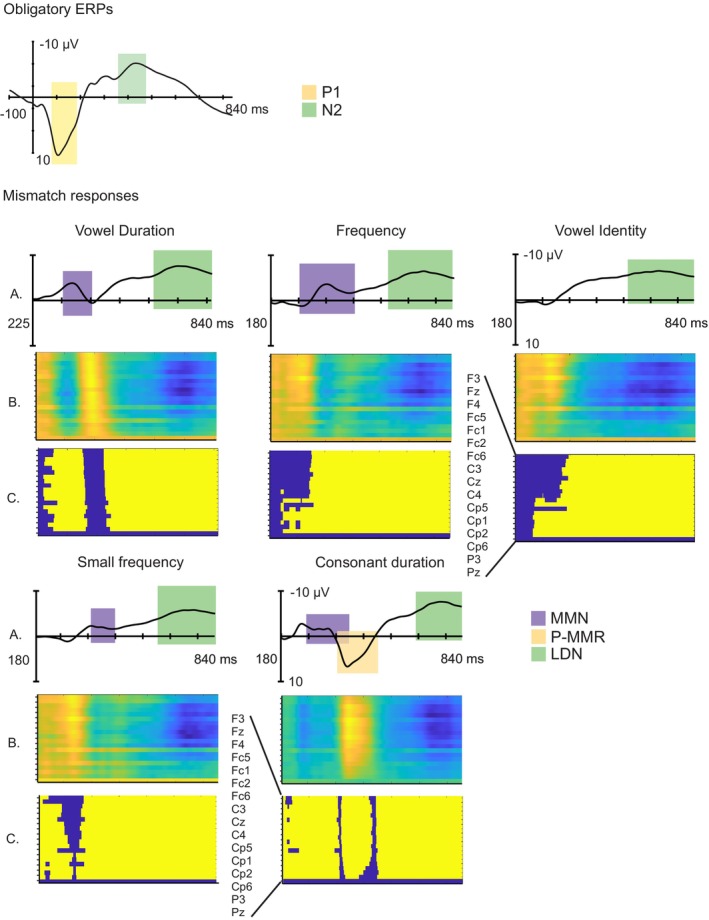
Auditory ERPs in the whole sample at 4–5 years. Top: Obligatory ERPs (P1 and N2) to the standard pseudoword /tata/ in the whole sample, averaged over the large region of interest (ROI) with baseline at stimulus onset (latency in ms, amplitude in μV). Colorful bars illustrate the time windows used for searching the individual peak latencies (yellow for P1, green for N2). Illustration for the auditory ERPs at 28 months can be found in Virtala et al. (2022). Bottom: (A) Mismatch responses (MMRs) mismatch negativity (MMN), positive mismatch response (P‐MMR), and late discriminative negativity (LDN) calculated from deviant minus standard subtraction waveforms and plotted from deviance onset (latency in ms, amplitude in mV), elicited by the vowel duration (upper left), frequency (upper middle), vowel identity (upper right), small frequency (bottom left), and consonant duration (bottom middle) deviants in the whole sample at the large ROI. Colorful bars on top of the MMRs illustrate the time windows used for searching the individual peak latencies. (B) The colorful panels show the deviant minus standard ERP responses indicating the MMRs (*y* axis is an arbitrary amplitude scale with positive values illustrated with warm colors, negative values with cold colors) at all electrodes included in the analysis (as labeled in the 28mo Panel C) from the deviance onset onwards (*x* axis with the same scale as for the MMRs). (C) The panels show time ranges (*x* axis with the same scale as for the MMRs) of statistically significant deviant–standard difference (as yellow squares) as determined by cluster‐based mass permutation tests.

At 4–5 years, the standard /tata/ stimulus elicited a two‐peaked complex consisting of a broad P1 that reached its maximum amplitude between 100 and 150 ms, followed by a N2 deflection peaking at around 400 ms. The elicitation of the obligatory ERPs is illustrated in Figure 2, and mean amplitudes and peak latencies are listed in Table 3.

**TABLE 3:** Mean amplitudes (in μV, with standard deviation, SD, in parentheses), peak latencies, and search windows of the obligatory elicited ERPs in the whole sample at birth (Newborn) and 28 months (28mo) at the large, left, and right regions of interest (ROIs).

	Mean amplitude, μV (SD)			Peak latency, ms (SD)	Peak latency *N*
	Large ROI	Left ROI	Right ROI		
28mo *N* = 146					
P1	8.10 (2.71)	8.06 (2.82)	8.17 (2.80)	132 (17.43)	
N2	−4.78 (3.11)	−4.58 (3.07)	−5.08 (3.27)	408 (37.22)	
Duration–MMN	−1.55 (3.38)	−1.52 (3.46)	−1.67 (3.49)	352 (23.25)	108
Frequency–MMN	−3.35 (3.80)	−3.24 (3.78)	−3.42 (4.10)	389 (40.78)	143
Duration–P‐MMR	2.34 (3.65)	2.35 (3.77)	2.20 (3.75)	422 (30.96)	138
Vowel–P‐MMR	2.86 (3.19)	2.94 (3.33)	2.71 (3.28)	357 (36.12)	136
Duration–LDN	−4.33 (3.54)	−4.08 (3.85)	−4.65 (3.59)	738 (46.27)	141
Frequency–LDN	−6.04 (3.79)	−6.26 (4.05)	−6.05 (3.79)	741 (47.26)	140
Vowel–LDN	−3.57 (3.82)	−3.43 (4.01)	−3.70 (3.79)	735 (50.89)	135
4.5yo *N* = 143					
P1	10.81 (3.03)	10.87 (2.99)	10.70 (2.88)	115 (14.84)	
N2	−6.62 (3.78)	−6.43 (3.91)	−6.79 (3.81)	420 (42.49)	
Duration–MMN	−3.53 (2.57)	−3.61 (2.63)	−3.64 (2.74)	337 (14.95)	131
Frequency–MMN	−3.97 (3.51)	−3.96 (3.73)	−4.15 (3.70)	390 (26.92)	136
Small frequency–MMN	−2.23 (3.76)	−2.15 (3.93)	−2.36 (3.98)	408 (28.28)	121
Consonant duration–MMN	−2.53 (2.54)	−2.51 (2.63)	−2.70 (2.68)	345 (26.95)	125
Consonant duration–P‐MMR	6.88 (4.19)	7.28 (4.44)	6.57 (4.59)	442 (17.34)	141
Duration–LDN	−7.98 (3.20)	−8.02 (3.34)	−8.15 (3.25)	736 (41.97)	138
Frequency–LDN	−7.14 (2.98)	−7.18 (3.18)	−7.36 (2.99)	724 (45.82)	141
Vowel–LDN	−6.87 (2.84)	−6.83 (2.98)	−7.25 (2.97)	702 (63.91)	141
Small frequency–LDN	−6.13 (3.04)	‐ 6.28 (3.14)	−6.31 (3.12)	740 (46.31)	141
Consonant duration–LDN	−8.15 (3.39)	−8.06 (3.80)	−8.42 (3.65)	752 (38.09)	133

*Note:* (1) Peak latency was searched separately from each individual and therefore the data contain missing values. Sample sizes available for each response at each age group are reported in the column “Peak latency *N*.” (2) The electrodes included in the large ROI are F3, Fz, F4, C3, Cz, C4, Fc1, Fc2, Fc5, and Fc6. The electrodes included in the left and right ROIs are F3, C3, Fc1, and Fc5 and F4, C4, Fc2, and Fc6, respectively.

### 3.2 Mismatch Responses in the Whole Sample at the Age of 4–5 Years

At 4–5 years, both the vowel duration, frequency, and small frequency deviants elicited a MMN followed by a LDN, whereas the vowel identity deviant produced only a broad LDN. Finally, the consonant duration deviant evoked a three‐peaked MMN–P‐MMR–LDN complex. The MMRs, deemed significant by the mass permutation tests, are illustrated in Figure 2, and mean amplitudes and peak latencies are listed in Table 3.

### 3.3 Maturation of Obligatory ERPs in the Whole Sample From 28 Months to 4–5 Years

**FIGURE 3:**
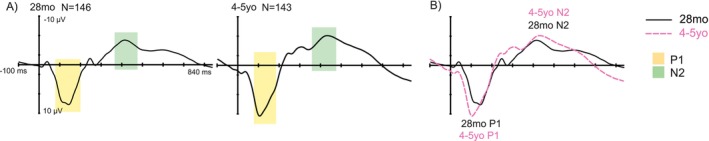
Maturation of the obligatory auditory event‐related potentials, P1 and N2. (A) ERP waveforms in the whole sample at 28 months (28mo) and 4–5 years (4–5yo), averaged over the large regions of interest (ROIs) with baseline at stimulus onset (latency in ms, amplitude in μV). Colorful bars illustrate the time windows used for searching the individual peak latencies (yellow for P1, green for N2). (B) ERP waveforms illustrating ERP maturation at 28mo (black, solid line) and 4–5yo (pink, dashed line), at the large ROIs, with baseline at stimulus onset.

The maturation of obligatory ERPs from 28 months to 4–5 years was evaluated using LMMs and is illustrated in Figure 3. A summary of the statistically significant effects is provided in Table 4. Complete statistics of the LMMs are provided in Table S1.

**TABLE 4:** Summary of the statistically significant (*p* < 0.05) main results of the linear mixed model (LMM) analyses for mean amplitudes (AMPL) and peak latencies (LAT) of both obligatory ERPs (P1 and N2) and MMRs (MMN, P‐MMR, and LDN) with the effect of time (age in months) and deviant type (only for MMRs; vowel duration, vowel identity, and/or bug frequency or all three) as fixed factors. Main effects of deviant type are not reported.

Age	AMPL/LAT	Effect	*p*	Description
P1				
28 months to 4.5 years	AMPL	Time	< 0.001	Larger P1 at 4.5 years than at 28 months
	LAT	Time	< 0.001	Earlier P1 at 4.5 years than at 28 months
N2				
28 months to 4.5 years	AMPL	Time	< 0.019	Larger N2 at 4.5 years than at 28 months
MMN				
28 months to 4.5 years	AMPL	Time	< 0.001	Larger MMN at 4.5 years than at 28 months
	AMPL	Time × deviant	< 0.001	Effect of time only for the vowel duration deviant
LDN				
28 months to 4.5 years	AMPL	Time	< 0.001	Larger LDN at 4.5 years than at 28 months
	AMPL	Time × deviant	< 0.001	Larger effect of time for the vowel duration and vowel identity deviants
28 months to 4.5 years	LAT	Time	< 0.001	Earlier LDN at 4.5 years than at 28 months
	LAT	Time × deviant	< 0.001	Effect of time only for the big frequency and vowel identity deviants

LMMs revealed that the P1 was significantly larger and earlier at 4–5 years than at 28 months (main effect of time for amplitude: *F*(1, 139) = 34.73, *p* < 0.001; main effect of time for latency: *F*(1, 118) = 34.21, *p* < 0.001). In addition, the N2 was significantly larger at 4–5 years than at 28 months (*F*(1, 145) = 5.59, *p* < 0.001), with no changes observed for its latency. When hemispheric lateralization was incorporated into the LMM, no significant main or interaction effects of hemisphere were found.

### 3.4 Maturation of MMRs in the Whole Sample From 28 Months to 4–5 Years

**FIGURE 4:**
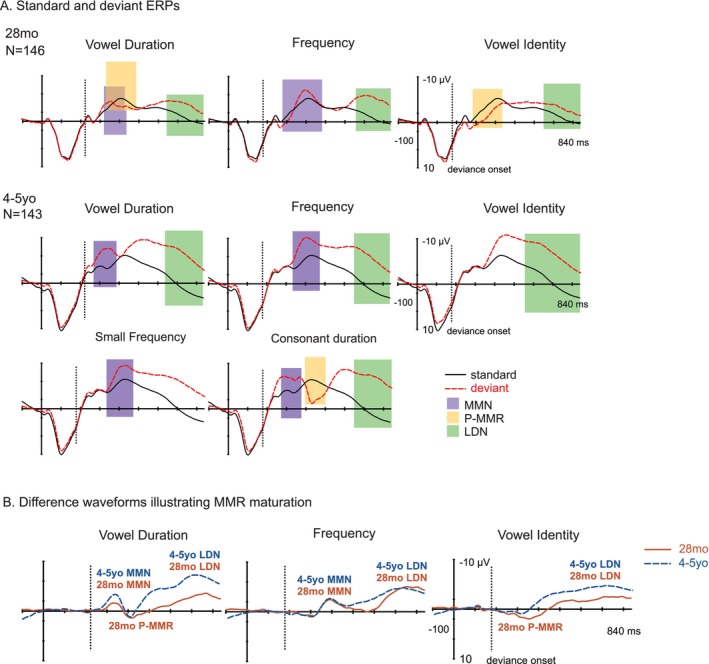
Maturation of the mismatch responses (MMRs) mismatch negativity (MMN), positive mismatch response (P‐MMR), and late discriminative negativity (LDN). (A) Standard (black, solid line) and deviant (red, dashed line) event‐related potentials (ERPs) to vowel duration (left), frequency (middle), and vowel identity deviants (right) in the whole sample at 28 months (28mo, top) and 4–5 years (4–5yo, bottom), depicted at the large regions of interest (ROIs) with baseline at stimulus onset (latency in ms, amplitude in mV). Colorful bars illustrate the time windows used for searching the individual peak latencies. (B) Subtraction waveforms illustrating MMR maturation at 28mo (red, solid line) and 4–5yo (blue, dashed line) at the large ROIs (as specified in Panel A), with baseline at deviance onset (marked with the vertical dashed line).

Maturational changes were analyzed from responses that were elicited at both age points by the same deviants. This included (1) MMN responses elicited by the vowel duration and frequency deviants and (2) LDN responses elicited by the vowel duration, frequency, and vowel identity deviants. The P‐MMRs elicited by the vowel duration and vowel identity deviants at 28 months were no longer significant at 4–5 years, and their maturation was thus not investigated with LMMs. The maturation of the MMRs is illustrated in Figure 4, and a summary of the statistically significant effects is provided in Table 4. Complete statistics of the LMMs are provided in Table S1.

MMN amplitudes significantly grew with age (main effect of time: *F*(1, 460) = 146.74, *p* < 0.001). Additionally, a significant time × deviant interaction was observed (*F*(1, 393) = 96.65, *p* < 0.001) and post hoc tests indicated that the effect was driven by the vowel duration MMN (*p* < 0.001 vs. *p* = 0.058 for the frequency MMN). No main or interaction effects of time were found for MMN latencies.

The LDN amplitudes also significantly grew with age (main effect of time: *F*(1, 747) = 30.01, *p* < 0.001), and the LDN latencies decreased with age (main effect of time: *F*(1, 736) = 28.20, *p* < 0.001). The latency analysis also yielded a significant time × deviant interaction (*F*(2, 630) = 7.01, *p* < 0.001), and the corresponding post hoc tests revealed that while LDNs in response to the frequency and the vowel identity deviants were significantly earlier at 4–5 years than at 28 months (frequency LDN: *p* = 0.001; vowel identity: *p* < 0.001), no changes in latency were found for the vowel duration deviant (*p* = 0.67).

When hemispheric lateralization (left vs. right ROI) was added to the LMMs, no significant main or interaction effects of hemisphere were found, and therefore, hemisphere was not included in the final models.

### 3.5 Effects of Dyslexia Risk on the Elicitation and Maturation of Auditory ERPs

**FIGURE 5:**
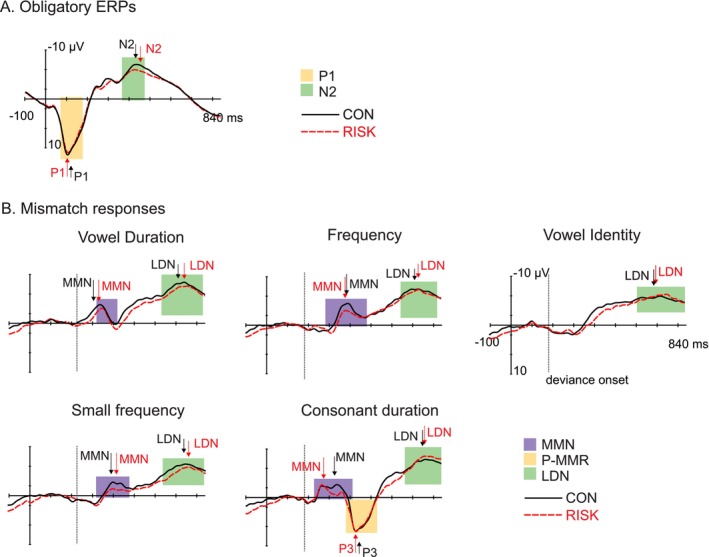
Auditory ERPs in the high‐risk and control subgroups at 4–5 years. (A) Obligatory ERPs (P1 and N2) to the standard pseudoword /tata/ in the dyslexia‐risk (RISK, red dashed line, red font) and control (CON, black solid line, black font) subgroups at birth (0mo, top), 6 months (6mo, middle), and 28 months (28mo, bottom), depicted at the large regions of interest (ROIs), with baseline correction at stimulus onset (dashed vertical line; latency in ms, amplitude in mV). Colorful bars illustrate the time windows used for searching the individual peak latencies (yellow for P1, green for N2), and arrows mark the mean peak latencies in each group (red = RISK, black = CON). (B) Mismatch responses (MMRs) mismatch negativity (MMN), positive mismatch response (P‐MMR), and late discriminative negativity (LDN) to duration (left), frequency (middle), and vowel deviants (right) in the dyslexia‐risk (RISK, red dashed line, red font) and control (CON, black solid line, black font) subgroups at 4–5 years, depicted at the large ROI, with baseline correction at deviance onset (dashed vertical line; latency in ms, amplitude in mV). Colorful bars illustrate the time windows used for searching the individual peak latencies, and arrows mark the mean peak latencies in each group (red and dashed = RISK, black and solid = CON).

ERP waveforms in the control and high‐risk subgroups are illustrated in Figure 5. Complete statistics for the final LMMs are provided in Table S2, and for the ANOVAs in Tables S3 and S4.

For the obligatory ERPs at 4–5 years, neither the one‐way ANOVAs nor the LMMs revealed significant effects of dyslexia risk on the amplitudes or latencies of the P1 or N2 components or on their maturation from 28 months to 4–5 years (Figure 5A). When hemispheric lateralization was added to the statistical analysis, no statistically significant interaction effects of hemisphere and risk were found.

For the MMRs at 4–5 years, one‐sample *t*‐test statistics of group‐wise response significance are reported in Table 5, and MMRs in the control and high‐risk subgroups are illustrated in Figure 5. All the MMRs elicited in the whole sample were also significantly elicited in both the control and high‐risk groups. The RM‐ANOVA on MMN amplitudes at 4–5 years revealed significantly larger amplitudes in controls than in at‐risk children (main effect of risk: *F*(1, 67) = 4.37, *p* = 0.023, Figure 5B and Table 5). No significant risk × deviant interaction was observed, suggesting that the effect of dyslexia risk was consistent across all deviant types. Furthermore, no group differences were observed in MMN latencies or in the amplitudes and latencies of the remaining MMRs. Similarly, when dyslexia risk was included as a factor in the LMMs, no significant effects were found on the maturation of MMRs from 28 months to 4–5 years. Finally, when hemispheric lateralization was added to the ANOVAS or LMMs, no statistically significant interaction effects of hemisphere and risk were found on the MMRs at 4–5 years or on their maturation from 28 months to 4–5 years. As dyslexia risk had an effect on the MMNs at 4–5 years, the maturation of the MMRs from 28 months to 4–5 years is separately illustrated for the control group only in Figure S1, showing similar maturational patterns as in the whole sample analysis.

**TABLE 5:** Mismatch negativity (MMN), positive mismatch response (P‐MMR), and late discriminative negativity (LDN) mean amplitudes (Mean ampl, in μV, with standard deviation SD in parentheses), peak latencies (in ms from deviance onset, standard deviation in parentheses), and one‐sample *t* statistics of mean amplitudes in the risk (RISK) and control (CON) subgroups at the large regions of interest (ROIs). Statistically significant mean amplitudes (after corrections) are in bold, and its significance level is indicated with asterisks (*p < 0.05, **p < 0.01, ***p < 0.001).

	Mean ampl, μV (SD)	*t* (df), *p*	Peak latency, ms (SD)	Peak latency *N*	Mean ampl, μV (SD)	*t* (df), *p*	Peak latency, ms (SD)	Peak latency *N*
4–5 years old (20 tests, Bonferroni‐corrected criterion *p* = 0.0025)								
	CON *N* = 32				RISK *N* = 30			
Duration–MMN	−3.60 (2.26)***	−15.06 (30), *p* < 0.000	108.13 (16.94)	30	−2.80 (2.56)***	−5.994 (29), *p* < 0.000	114.63 (15.44)	27
Frequency–MMN	−4.75 (4.08)***	−6.58 (31), *p* < 0.000	205.73 (22.26)	30	−3.27 (4.01)***	−4.446 (29), *p* < 0.000	208.00 (23.70)	27
Small frequency–MMN	−3.14 (4.14)***	−4.28 (31), *p* < 0.000	228.82 (25.15)	29	−1.58 (3.30)**	−2.61 (29), *p* = 0.007	222.58 (27.34)	24
Consonant duration–MMN	−3.10 (2.62)***	−6.69 (31), *p* < 0.000	162.96 (24.40)	27	−1.70 (2.24)***	−4.16 (29), *p* < 0.000	165.92 (30.58)	25
Consonant duration–P‐MMR	7.14 (5.63)**	7.17 (31), *p* = 0.001	265.75 (19.11)	32	7.21 (2.85)***	13.83 (29), *p* < 0.000	262.27 (15.47)	30
Duration–LDN	−8.79 (3.25)***	−9.00 (31), *p* < 0.000	507.87 (39.64)	30	−8.68 (3.07)***	−15.449 (29), *p* < 0.000	564 (45.12)	29
Frequency–LDN	−8.15 (3.67)***	−12.55 (31), *p* < 0.000	545.63 (48.28)	32	−7.68 (2.30)***	−18.279 (29), *p* < 0.000	542.27 (42.27)	30
Vowel–LDN	−6.36 (2.60)***	−13.61 (30), *p* < 0.000	514.81 (73.53)	32	−7.08 (2.01)***	−19.226 (29), *p* < 0.000	524.15 (60.06)	29
Small frequency–LDN	−6.88 (3.66)***	−10.62 (31), *p* < 0.000	569.41 (47.42)	31	−6.38 (2.31)***	−15.08 (29), *p* < 0.000	554.53 (40.42)	30
Consonant duration–LDN	−8.52 (3.77)***	−12.78 (31), *p* < 0.000	574.12 (47.15)	31	−9.11 (3.13)***	−15.92 (29), *p* < 0.000	577.86 (29.23)	28

## 4 Discussion

Despite the critical role of the preschool period in the development of literacy and the underlying neural processing of speech sounds, the elicitation and maturation of speech‐evoked ERPs and MMRs during this stage have rarely been studied. By introducing five different changes in a pseudoword stream, we aimed to determine the typical morphology of obligatory ERPs and MMRs to speech sounds at the age of 4–5 years in the large DyslexiaBaby sample. As expected, the standard stimulus elicited a clear P1–N2 complex. In addition, the vowel duration, frequency, and small frequency deviants elicited an MMN followed by an LDN. The vowel identity deviant elicited only a broad LDN response. Finally, the consonant duration deviant elicited a three‐peaked MMN–P‐MMR–LDN complex. Additionally, this work aimed to determine the maturation of these responses between 28 months and 4–5 years of age by utilizing the previously reported data from the same children at 28 months. The P1 component increased in amplitude and decreased in latency between these two age points, while the N2 only increased in amplitude. The MMN exhibited an amplitude increase in response to the vowel duration deviant, with no significant changes in latency. The P‐MMR, which was present at 28 months to vowel duration and vowel identity deviants, was no longer observable at the age of 4–5 years. The LDN increased in amplitude across all deviant types and decreased in latency in response to the frequency and vowel identity deviants.

The current study also aimed to address the impact of familial dyslexia risk on speech‐elicited ERPs and MMRs at 4–5 years of age, and on their maturation from 28 months to 4–5 years. At 4–5 years, the MMRs that were statistically significant in the whole sample also reached significance within both the control and dyslexia‐risk subgroups. The children in the dyslexia‐risk group showed consistently smaller MMN amplitudes across deviant types compared with controls. No effects of dyslexia risk were observed on the rest of the MMRs, on the obligatory ERPs, or on their maturation from 28 months to 4–5 years.

Overall, this study validates and further extends previous findings on ERPs and MMRs to speech sounds during the preschool stage with a larger sample size (*N* = 143–146 depending on the age point). In addition, it demonstrates that atypical speech‐sound discrimination, as indexed by a reduced MMN, is associated with increased risk for dyslexia in preschool children. These results may serve as a foundational reference for future studies investigating the effects of developmental conditions on speech‐sound processing and language and literacy development.

### 4.1 Elicitation of ERPs to Speech Sounds at 4–5 Years

Based on previous reports, we expected a two‐peaked pattern in obligatory ERPs at 4–5 years, consisting of a broad positivity (P1) followed by a negativity (N2; Shafer et al. 2010, 2000; Gomot et al. 2000; Linnavalli et al. 2018). The present results are consistent with this pattern, validating earlier findings with a larger sample size (*N* = 143, compared with *N* = 12–74 in prior studies). Overall, our findings suggest that the P1 and N2 remain the predominant obligatory ERP components elicited by speech sounds at the preschool age.

The change‐detection waveforms were primarily characterized by negative peaks (MMN and LDN), aligning with our hypotheses and with previous findings in both preschoolers (Ceponiene et al. 2002; Korpilahti et al. 2001; Linnavalli et al. 2018) and school‐aged children (Hommet et al. 2009). Although both of these MMRs reflect aspects of auditory change detection, they appear to have distinct neural generators (Ceponiene et al. 2005; Hommet et al. 2009), and therefore, they are interpreted to have different functional significances. Whereas the MMN seems to reflect genuine deviance detection, the LDN was suggested to be particularly associated with speech or higher order processing of stimuli (e.g., Bishop et al. 2011; Kuuluvainen, Alku, et al. 2016). The P‐MMR, in turn, was almost absent at 4–5 years, emerging only for the consonant duration deviant, and may reflect processes other than genuine deviance detection (further discussed in Section 4.4). These results are in line with the idea that this response is no longer a prevalent or predominant index of auditory change detection at the preschool stage (Shafer et al. 2011; Cheng et al. 2013, 2015).

### 4.2 Maturation of ERPs to Speech Sounds From 28 Months to 4–5 Years

Consistent with our hypotheses and previous research, both the P1 and the N2 increased in amplitude between 28 months and 4–5 years. Also, P1 latency decreased during this period. These developmental changes continue the trajectory observed in this sample between birth and 28 months (Navarrete‐Arroyo et al. 2025), indicating a gradual increase in obligatory ERP amplitudes during the first years of life. For the ERP latencies, results were less consistent in the earlier phases of the follow‐up, with P1 showing decreased and N2 increased latencies with increasing age (Navarrete‐Arroyo et al. 2025). Based on previous research, it is expected that these responses reach their maximum amplitudes in early childhood (6–8 years of age), before starting to diminish (Ponton et al. 2002; Sussman et al. 2008). Future follow‐ups within the DyslexiaBaby study will assess whether this maturational pattern persists beyond 4–5 years in the current cohort and how the adult‐like P2–N1 complex starts emerging between the child–P1–N2 complex (Shafer et al. 2015). Functionally, the increment of the P1 and the N2 amplitudes could be linked with an increased synaptic density and connectivity of the auditory system (Sussman et al. 2008; Yvert et al. 2005). In addition, observing shortening latencies for the P1 with increasing age suggests an ongoing maturation of white matter and increasing myelinization in auditory pathways and early cortical areas (Ponton et al. 2000; Moore and Linthicum 2007).

The maturation of the MMRs between 28 months and 4–5 years compared with that of the obligatory ERPs was notably more profound, with large changes in response morphology. Firstly, the maturation was characterized by increasing amplitudes of the MMN and the LDN, consistent with previous reports (Shafer et al. 2010; Choudhury and Benasich 2011; Lee et al. 2012; Morr et al. 2002) and developmental trends observed in the DyslexiaBaby sample (Virtala et al. 2022). However, the MMN increased in response to the vowel duration deviant, but not to the frequency deviant, although a near‐significant trend was observed. This suggests that discrimination of large pitch changes matures earlier in development, leaving little room for further amplitude increases. This interpretation is further supported by the morphology of the (large) frequency MMR at 28 months: It was the only deviant type that demonstrated a mature pattern of two negative MMRs, MMN, and LDN, already at that age (Virtala et al. 2022). The findings for the LDN align with previous studies reporting its growth during the first 10 years of life (Shafer et al. 2010), after which it should start to diminish (Cheour et al. 2000; Linnavalli et al. 2018). The LDN amplitude increase likely reflects the refinement of higher order auditory processing, including attentional reorientation and memory‐based stimulus evaluation (Bishop et al. 2011).

Secondly, as hypothesized, the MMR maturation was characterized by the P‐MMRs largely vanishing by 4–5 years. The P‐MMRs elicited at 28 months in response to the vowel duration and vowel identity deviants were no longer observed at 4–5 years. This pattern supports the view that P‐MMRs reflect immature auditory discrimination mechanisms, which are gradually supplanted by the negative responses (i.e., enhanced MMN and LDN amplitudes) as the auditory system matures (He et al. 2009; Trainor 2012). Taken together, these findings indicate that a typical development of auditory change detection in the preschool years is primarily reflected by increasing amplitudes of the MMN and LDN and disappearance of the infant‐P‐MMR. However, it is important to note that these findings concern the whole DyslexiaBaby sample, where a majority of the children are at familial risk for dyslexia. Therefore, to make conclusions on typical auditory processing, we determined the effects that dyslexia risk had on the obligatory ERPs, change‐related MMRs, and their maturation from 28 months to 4–5 years.

### 4.3 Effects of Dyslexia Risk on ERPs and Their Maturation During the Preschool Stage

We obtained no effects of familial dyslexia risk on the obligatory ERPs at 4–5 years or their maturation. Similarly, no dyslexia‐risk effects were visible on the LDNs and P‐MMRs at 4–5 years or on the maturation of all the MMRs. Thus, despite the characteristics of the present sample, the morphology and maturation of these responses can largely be interpreted to represent age‐typical patterns (Figure 5). However, for the MMNs elicited at 4–5 years, effects of familial dyslexia risk were consistently observed as reviewed below.

The diminished MMN to speech sounds in the high‐risk group at 4–5 years was an expected result based on previous MMN findings in small children, particularly in response to phoneme changes (Leppänen et al. 2002; van Zuijen et al. 2012; Plakas et al. 2013; Thiede et al. 2019, with partly overlapping newborn data to the present study). Similar reductions in MMN responses have also been reported in adults with dyslexia (e.g., Schulte‐Körne et al. 2001). This reduced MMN could be interpreted as deficient speech‐sound processing abilities in dyslexia risk, consistent with the phonological deficit theory (Peterson and Pennington 2015; Vellutino et al. 2004). In line with this, we previously found that 28‐month‐old children from the DyslexiaBaby sample had atypical P‐MMRs to speech‐sound changes (Virtala et al. 2022). Hence, at both follow‐up points, the effects of dyslexia risk were seen in the most prominent change‐detection response of that age (MMN at preschool age, P‐MMR in infancy and early childhood). In line with the general maturational trends of the MMRs, the children with larger P‐MMRs coupled with smaller MMNs and LDNs at 28 months also exhibited poorer prereading skills during the preschool stage (Navarrete‐Arroyo et al. 2024). While MMR maturation did not seem to be affected by dyslexia risk in the present study, our earlier report did show some effects of dyslexia risk on how these MMRs changed between birth and 28 months of age (Virtala et al. 2022). Group differences were mainly seen in the LDNs that were more prominent in the risk than control group at 6 months, increased in amplitude and latency more between 6 and 28 months in the control than risk group, and ended up being somewhat more prominent in the control than risk group at 28 months (Virtala et al. 2022). It is noteworthy that these maturational differences between the risk and control groups that seemed to take place by 28 months did not result in group effects anymore between 28 months and 4–5 years. Future investigations in the DyslexiaBaby project will reveal how the 4‐ to 5‐year MMRs mature further and show associations with both familial dyslexia risk and emerging literacy.

No effect of dyslexia risk was observed on the elicitation of obligatory ERPs. Although some differences may be expected, this result aligns with earlier findings from the DyslexiaBaby study where only minor effects of dyslexia risk were seen on the obligatory responses in infancy and at 28 months (Navarrete‐Arroyo et al. 2025). Also previously, the influence of dyslexia and familial risk on obligatory ERPs has been inconsistent, with reported effects varying considerably across studies (e.g., Sharma et al. 2006; Stefanics et al. 2011; Lachmann et al. 2005; Bonte et al. 2007). Notably, this finding of lacking group effects on the obligatory ERPs differs from the here‐reported effects of dyslexia risk on MMRs. This suggests that auditory deficits in dyslexia risk may stem primarily from impaired discrimination of speech sounds rather than sound encoding. Neural speech encoding, reflected by obligatory ERPs, involves early‐stage processing of basic sound properties, which is robust already in infancy (Ceponiene et al. 2005, 2008). In contrast, auditory discrimination relies on higher order processing that integrates more sensory information (Näätänen et al. 2019), making it more vulnerable to developmental disorders like dyslexia. Taken together, the present study thus suggests that MMRs might hold greater potential as neural markers of dyslexia than obligatory ERPs. Nevertheless, caution is warranted when considering individual‐level predictions. Variability in MMR polarity and amplitude across children, along with ongoing neural maturation, limits the reliability of ERPs for single‐subject interpretation. Instead, they provide valuable insight into neural mechanisms underlying dyslexia and its development in a general level.

The present study did not find any atypical lateralization of the MMRs or the obligatory ERPs in the dyslexia high‐risk group, thus providing no support for the suggested left‐hemispheric dysfunctions in dyslexia (Kuuluvainen, Alku, et al. 2016; Leppänen et al. 2002; van Leeuwen et al. 2007; Khan et al. 2011). This was somehow expected, as no notable lateralization differences were found in this sample from birth to 28 months (Navarrete‐Arroyo et al. 2025; Virtala et al. 2022). The ongoing follow‐up of the DyslexiaBaby cohort at school age, incorporating structural and functional magnetic resonance imaging in addition to ERPs, will allow for notably more detailed examinations of hemispheric contributions to auditory processing abnormalities associated with dyslexia.

Previous studies have indicated that auditory processing deficits are often the most pronounced at birth but may attenuate or be compensated for during early development, becoming less detectable by later childhood (Galaburda et al. 2006; Hämäläinen et al. 2013). The present finding of a persistent MMN reduction at the preschool stage, coupled with our earlier report of persistent P‐MMR abnormalities in early childhood (Virtala et al. 2022), implies that these early auditory processing difficulties may not resolve by preschool age. Instead, they may continue to manifest as subtle deficits in speech‐sound discrimination that can still be detected beyond infancy and toddlerhood with the use of age‐appropriate and sufficiently sensitive paradigms. Importantly, the inclusion of a larger set of deviants in the paradigm at 4–5 years may have contributed to revealing these group differences, suggesting that increasing the range and complexity of tested contrasts may enhance the sensitivity of MMR paradigms in detecting risk‐related auditory processing difficulties. Future studies within the DyslexiaBaby cohort at older ages will help to determine whether these deficits persist or resolve by school age.

### 4.4 Effects of Deviant Type on MMR Elicitation

The present study utilized a speech‐sound paradigm consisting of five different types of change embedded in a pseudoword stream at 4–5 years of age. Based on results from the previous follow‐up of the DyslexiaBaby sample with three of these change types included (e.g., Virtala et al. 2022), we expected that different change types may elicit different MMR patterns, maturational trajectories, and effects of dyslexia risk. This was assumed to be partly due to differences in acoustic salience, as subtle changes may elicit immature MMRs at early ages and be especially sensitive to dyslexia‐related differences in adults (Cheng et al. 2013, 2015; Cheng and Lee 2018; Baldeweg et al. 1999). However, the investigation of the differences between deviants was exploratory and not among the primary aims of the present study.

Following this line of thought, the vowel duration and frequency deviants may be the most acoustically salient of the five, providing an explanation for its most mature response pattern at 4–5 years (a large MMN followed by a broad LDN). In contrast, the vowel identity deviant may be particularly challenging, as we only observed it to elicit a broad LDN. This more immature MMR in response to the vowel identity deviant compared with the other deviant types was already observed at 28 months, when it elicited a P‐MMR followed by an LDN, with no MMN (Virtala et al. 2022). A possible explanation for it is that the vowel identity deviant presented an acoustically subtle phonemic contrast, specifically challenging for this sample of mostly dyslexia‐risk children, while the rest of the deviants rely on manipulation of basic sound features (duration or pitch).

Notably, the paradigm at 4–5 included two new deviant types with the intention of increasing the sensitivity to dyslexia risk and general demand level of the experiment as children grow older. The effects of dyslexia risk did not appear to depend on the deviant type but were instead observed consistently across them. Nevertheless, the small frequency deviant elicited a similar MMN–LDN pattern as the frequency deviant. However, the MMN amplitude was significantly smaller (−2.23 vs. −3.97 μV), suggesting that the smaller frequency change was more challenging to discriminate irrespective of dyslexia‐risk status. The consonant duration deviant elicited a more complex response pattern (MMN–P‐MMR–LDN) compared with the rest of deviants. This pattern may partly reflect onset/offset responses to the abrupt closure and release in the deviant, rather than purely discrimination‐related activity. The presence of an MMN nevertheless suggests preattentive detection of the contrast, consistent with the perceptual difficulty of consonant quantity detection in early development (Richardson et al. 2004; Ylinen et al. 2005; Nakai et al. 2009). Moreover, the two‐peaked MMN morphology in response to the consonant duration deviant may reflect its specific temporal structure, which involves an initial omission of the expected syllable onset followed by its delayed occurrence.

Overall, these results highlight the importance of carefully selecting deviant features when designing auditory paradigms. Whenever possible, future studies should employ multifeature designs that include several deviant types, enabling a broader assessment of auditory discrimination within a short recording time (Näätänen et al. 2004).

### 4.5 Limitations

When interpreting the results of this study, it is important to consider the overrepresentation of children at risk for dyslexia in the sample. This sampling characteristic enhances the study's relevance for understanding dyslexia‐specific developmental trajectories but may limit generalizability to typically developing populations. Based on the dyslexia risk versus control group comparisons in this study and our previous publication (Virtala et al. 2022), the overrepresentation of the dyslexia‐risk children diminishes the MMNs at 4–5 years and diminishes the phoneme–P‐MMRs and increases the duration–P‐MMRs at 28 months. No direct effects of dyslexia risk on the MMR maturation were observed. However, it is possible that in a larger sample comprised of only control children, MMN would have been more pronounced at 4–5 years, and its maturation might have been more evident across different time points. Nevertheless, the elicitation and maturation of the MMRs between the two measurement points in the control group only show a pattern very similar to the whole sample, as visualized in Figure S1.

When interpreting the maturation of speech‐elicited ERPs from 28 months to 4–5 years, it is important to acknowledge the differences in the experimental paradigms, caused by the addition of two more deviant types to the paradigm at the 4‐ to 5‐year follow‐up point. First, the 28‐month paradigm's deviant presentation (minimum one standard between deviants) versus the 4‐ to 5‐year‐old's strict alternation (deviant–standard–deviant) may differentially affect refractory period recovery and attention modulation, potentially influencing ERP amplitudes. Second, the higher standard probability at 28 months than 4–5 years (71.4% vs. 50%) could enhance deviant detectability through stronger memory trace formation. These issues could in turn result in more pronounced MMRs at 28 months than 4–5 years. However, as both the MMN and LDN increased with age, this difference in standard probability has likely attenuated rather than intensified the obtained effects. On the other hand, the absence of the P‐MMR at 4–5 years might partly be explained by the deviants being “less surprising” and thus less attention catching at this age than at 28 months, at least if the P‐MMR is interpreted to be an immature P3a (Kushnerenko et al. 2013). Nevertheless, it is important to note that the interstimulus interval and deviant probability were kept constant between the 28‐month and 4‐ to 5‐year paradigms. Notably, previous research has shown that adult MMN amplitudes remain stable even when multiple deviant types are included, provided that stimulus parameters such as timing and probability are carefully controlled (Näätänen et al. 2004; Pakarinen et al. 2007, 2009).

Furthermore, while the EEG preprocessing pipelines at 28 months and 4–5 years shared core steps, some age‐appropriate adjustments were made to account for developmental differences in neural activity and artifact profiles. At 4–5 years, a stricter initial high‐pass filter (0.5 vs. 0.025 Hz) was applied to mitigate slow‐wave drift, followed by a separate 25‐Hz low‐pass filter applied post‐ICA to address residual high‐frequency noise, more prevalent in older children. In contrast, at 28 months, a 0.5‐ to 25‐Hz bandpass filter was applied before ICA. All the adjustments were made for the 4‐ to 5‐year data to achieve good data quality as effectively as the current software/toolboxes allow, based on the data characteristics from preschool‐aged children. Although these minor differences in the sequence of processing steps and artifact rejection thresholds could slightly influence data integrity and final signal quality, these adaptations were necessary to optimize data quality at each age. Moreover, key parameters (e.g., baseline correction windows, re‐referencing, epoch rejection criteria, interpolation limits, and ICA procedures) were harmonized and possible, increasing confidence that the observed ERP differences reflect maturation rather than methodological inconsistencies.

Finally, regarding the effects of dyslexia risk on MMRs elicitation, it should be noted that the observed reduced MMN in at‐risk children at group level reflects an association with dyslexia risk rather than an ability to predict outcomes in individual children. Variability in MMR polarity and amplitude across participants, along with ongoing neural maturation, limits the reliability of ERPs for single‐subject predictions. Future studies could combine ERPs with other risk indicators and refine analysis approaches to better understand early neural correlates of reading difficulties.

### 4.6 Conclusions

The present study, with its large‐scale and longitudinal design, provides a comprehensive characterization of speech‐elicited ERPs during the preschool years. At 4–5 years, neural speech‐sound encoding, as reflected by the obligatory ERPs, followed a P1–N2 pattern, while neural speech‐sound discrimination reflected by the MMRs was mainly characterized by a MMN followed by an LDN. During the maturation of these responses from 28 months to 4–5 years, the P1 increased in amplitude and decreased in latency, while the N2 increased in amplitude without latency changes. MMR maturation was characterized by an increase in MMN and LDN amplitudes and a decrease in LDN latency, while the P‐MMR observed at 28 months was no longer evident by 4–5 years. Notably, the present study also demonstrates dyslexia‐risk effects on neural speech‐sound discrimination at preschool age, with the risk group showing reduced MMNs across five deviant types at 4–5 years. In contrast, no group differences were found for the obligatory ERPs, suggesting that the auditory deficits associated with dyslexia are more tied to speech discrimination processes rather than basic sound encoding. This highlights how MMRs, and in particular the MMN, can provide insight into neural processes linked to dyslexia risk in preschool children.

## Author Contributions

**Sergio Navarrete‐Arroyo:** formal analysis, writing – original draft, visualization. **Peixin Nie:** formal analysis, writing – review and editing. **Paula Virtala:** supervision, writing – review and editing. **Teija Kujala:** conceptualization, supervision, writing – review and editing.

## Disclosure

During the preparation of this work, no generative AI or AI‐assisted technologies have been implemented.

## Conflicts of Interest

The authors declare no conflicts of interest.

## Supporting information


**Figure S1:** Subtraction waveforms illustrating MMR maturation in the control group between 28 months (red line) and 4–5 years old (blue line), at the large ROIs (F3, Fz, F4, C3, Cz, C4, Fc1, Fc2, Fc5, and Fc6), with baseline at deviance onset (marked with the vertical dashed line).

**Table S1:** Results of the linear mixed model (LMM) analyses for mean amplitudes (AMPL) and peak latencies (LAT) of both obligatory ERPs (P1 and N2) and MMRs (MMN, P‐MMR, and LDN) with the effect of time (age in months, mo) and deviant type (only for MMRs; vowel duration, vowel identity, and/or bug frequency or all three) as fixed factors. Main effects of deviant type are not reported.
**Table S2:** Results of the linear mixed model (LMM) analyses for mean amplitudes (AMPL) and peak latencies (LAT) of both obligatory ERPs (P1 and N2) and MMRs (MMN, P‐MMR, and LDN) with the effect of time (age in months, mo), deviant type (only for MMRs; vowel duration, vowel identity, and/or bug frequency or all three), and dyslexia risk (high risk vs. control) as fixed factors.
**Table S3:** Results of the one‐way ANOVAs testing the effect of dyslexia risk (high risk vs. control) on the mean amplitudes (AMPL) and peak latencies (LAT) of obligatory ERPs (P1 and N2).
**Table S4:** Results of the repeated‐measures ANOVAs for mean amplitudes (AMPL) and peak latencies (LAT) of MMRs (MMN, P‐MMR, and LDN) at 4–5 years, with deviant type (vowel duration, vowel identity, frequency, small frequency, and consonant duration) as repeated‐measures factor and with dyslexia risk (high risk vs. control) as between‐subjects factor.

## Data Availability

The aggregated data including the code used for data analysis are provided via Figshare repository (10.6084/m9.figshare.31047760). The raw data are available from the corresponding author upon request.
