# Supplementary material for: The Effects of Maturation and Dyslexia Risk on Neural Speech‐Sound Encoding and Discrimination at Preschool Stage
Source: Eur J Neurosci. 2026 Mar 8;63(5):e70450. doi: 10.1111/ejn.70450 (PMC12967738; doi:10.1111/ejn.70450)
Supplement: Supplementary file 2 — Table S1: Results of the linear mixed model (LMM) analyses for mean amplitudes (AMPL) and peak latencies (LAT) of both obligatory ERPs (P1 and N2) and MMRs (MMN, P‐MMR, and LDN) with the effect of time (age in months, mo) and deviant type (only for MMRs; vowel duration, vowel identity, and/or bug frequency or all three) as fixed factors. Main effects of deviant type are not reported. Table S2: Results of the linear mixed model (LMM) analyses for mean amplitudes (AMPL) and peak latencies (LAT) of both obligatory ERPs (P1 and N2) and MMRs (MMN, P‐MMR, and LDN) with the effect of time (age in months, mo), deviant type (only for MMRs; vowel duration, vowel identity, and/or bug frequency or all three), and dyslexia risk (high risk vs. control) as fixed factors. Table S3: Results of the one‐way ANOVAs testing the effect of dyslexia risk (high risk vs. control) on the mean amplitudes (AMPL) and peak latencies (LAT) of obligatory ERPs (P1 and N2). Table S4: Results of the repeated‐measures ANOVAs for mean amplitudes (AMPL) and peak latencies (LAT) of MMRs (MMN, P‐MMR, and LDN) at 4–5 years, with deviant type (vowel duration, vowel identity, frequency, small frequency, and consonant duration) as repeated‐measures factor and with dyslexia risk (high risk vs. control) as between‐subjects factor. [file EJN-63-0-s001.docx]

**Supplementary Table S1.**

Results of the linear mixed model (LMM) analyses for mean amplitudes (AMPL) and peak latencies (LAT) of both obligatory ERPs (P1 and N2) and MMRs (MMN, P-MMR, and LDN) with the effect of time (age in months, mo) and deviant type (only for MMRs; vowel duration, vowel identity, and/or bug frequency or all three) as fixed factors. Main effects of deviant type are not reported.

| Age | AMPL/LAT | Effect | *p* | F -value |
| --- | --- | --- | --- | --- |
| **P1** |  |  |  |  |
| 28mo – 4.5yo | AMPL | Time | <.001 | 34.732 |
|  | LAT | Time | <.001 | 34.210 |
| **N2** |  |  |  |  |
| 28mo – 4.5yo | AMPL | Time | <.019 | 5.598 |
|  | LAT | Time | .215 | 1.545 |
| **MMN** |  |  |  |  |
| 28mo – 4.5yo | AMPL | Time | <.001 | 146.744 |
|  | LAT | Time | .088 | 2.913 |
|  | AMPL | Deviant | <.001 | 29.341 |
|  | LAT | Deviant | <.001 | 432.907 |
|  | AMPL | Time x Deviant | <.001 | 96.657 |
|  | LAT | Time x Deviant | .557 | 0.344 |
| **LDN** |  |  |  |  |
| 28mo – 4.5yo | AMPL | Time | <.001 | 30.013 |
|  | LAT | Time | <.001 | 28.207 |
|  | AMPL | Deviant | <.001 | 53.950 |
|  | LAT | Deviant | <.001 | 11.005 |
|  | AMPL | Time x Deviant | <.001 | 30.698 |
|  | LAT | Time x Deviant | <.001 | 7.0169 |

**Supplementary Table S2.**

Results of the linear mixed model (LMM) analyses for mean amplitudes (AMPL) and peak latencies (LAT) of both obligatory ERPs (P1 and N2) and MMRs (MMN, P-MMR, and LDN) with the effect of time (age in months, mo), deviant type (only for MMRs; vowel duration, vowel identity, and/or bug frequency or all three), and dyslexia risk (high-risk vs. control) as fixed factors.

| Age | AMPL/LAT | Effect | *p* | F-value |
| --- | --- | --- | --- | --- |
| **P1** |  |  |  |  |
| 28mo – 4.5yo | AMPL | Time | <.001 | 33.332 |
|  | LAT | Time | <.001 | 52.910 |
|  | AMPL | Risk | .406 | 0.695 |
|  | LAT | Risk | .209 | 1.603 |
|  | AMPL | Time x Risk | .939 | 0.005 |
|  | LAT | Time x Risk | .844 | 0.038 |
| **N2** |  |  |  |  |
| 28mo – 4.5yo | AMPL | Time | .016 | 5.994 |
|  | LAT | Time | .183 | 1.807 |
|  | AMPL | Risk | .748 | 0.103 |
|  | LAT | Risk | .498 | 0.462 |
|  | AMPL | Time x Risk | .620 | 0.246 |
|  | LAT | Time x Risk | .458 | 0.553 |
| **MMN** |  |  |  |  |
| 28mo – 4.5yo | AMPL | Time | <.001 | 73.121 |
|  | LAT | Time | .079 | 3.1161 |
|  | AMPL | Deviant | <.001 | 15.859 |
|  | LAT | Deviant | <.001 | 267.835 |
|  | AMPL | Risk | .186 | 1.779 |
|  | LAT | Risk | .768 | 0.0871 |
|  | AMPL | Time x Deviant | <.001 | 54.831 |
|  | LAT | Time x Deviant | .475 | 0.510 |
|  | AMPL | Time x Risk | .823 | 0.049 |
|  | LAT | Time x Risk | .548 | 0.361 |
|  | AMPL | Deviant x Risk | .667 | 0.184 |
|  | LAT | Deviant x Risk | .026 | 5.021 |
|  | AMPL | Time x Deviant x Risk | .205 | 1.615 |
|  | LAT | Time x Deviant x Risk | .134 | 2.263 |
| **LDN** |  |  |  |  |
| 28mo – 4.5yo | AMPL | Time | <.001 | 2.263 |
|  | LAT | Time | .002 | 9.489 |
|  | AMPL | Deviant | <.001 | 39.588 |
|  | LAT | Deviant | <.001 | 9.314 |
|  | AMPL | Risk | .214 | 1.571 |
|  | LAT | Risk | .247 | 1.361 |
|  | AMPL | Time x Deviant | <.001 | 14.06 |
|  | LAT | Time x Deviant | .169 | 1.784 |
|  | AMPL | Time x Risk | .417 | 0.658 |
|  | LAT | Time x Risk | .836 | 0.042 |
|  | AMPL | Deviant x Risk | .045 | 3.114 |
|  | LAT | Deviant x Risk | .482 | 0.731 |
|  | AMPL | Time x Deviant x Risk | .866 | 0.143 |
|  | LAT | Time x Deviant x Risk | .046 | 3.107 |

**Supplementary Table S3.**

Results of the One-Way ANOVAs testing the effect of dyslexia risk (high-risk vs control) on the mean amplitudes (AMPL) and peak latencies (LAT) of obligatory ERPs (P1 and N2).

| Age | AMPL/LAT | Effect | *p* | F-value |
| --- | --- | --- | --- | --- |
| **P1** |  |  |  |  |
| 4.5yo | AMPL | Risk | .482 | 0.499 |
|  | LAT | Risk | .335 | 0.944 |
| **N2** |  |  |  |  |
| 4.5yo | AMPL | Risk | .940 | 0.005 |
|  | LAT | Risk | .695 | 0.155 |

**Supplementary Table S4.**

Results of the Repeated-Measures ANOVAs for mean amplitudes (AMPL) and peak latencies (LAT) of MMRs (MMN, P-MMR, and LDN) at 4-5 years, with deviant type (vowel duration, vowel identity, large frequency, small frequency, consonant duration) as Repeated-Measure factor and with dyslexia risk (high-risk vs. control) as Between-subjects factor.

| Age | AMPL/LAT | Effect | *p* | F-value |
| --- | --- | --- | --- | --- |
| **MMN** |  |  |  |  |
| 4.5yo | AMPL | Risk | .023 | 4.67 |
|  | AMPL | Risk x Deviant | .238 | 1.42 |
|  | LAT | Risk | .569 | 0.329 |
|  | LAT | Risk x Deviant | .372 | 1.05 |
| **P-MMR** |  |  |  |  |
| 4.5yo | AMPL | Risk | .917 | 0.010 |
|  | LAT | Risk | .313 | 1.036 |
| **LDN** |  |  |  |  |
| 4.5yo | AMPL | Risk | .687 | 0.163 |
|  | AMPL | Risk x Deviant | .574 | 0.727 |
|  | LAT | Risk | .880 | 0.603 |
|  | LAT | Risk x Deviant | .661 | 0.022 |
